# Association of Fat Mass and Obesity (FTO) rs9939609 Single Nucleotide Polymorphism (SNP) With Obesity and Type 2 Diabetes (T2D) in Healthy Young Adults in Kuwait

**DOI:** 10.7759/cureus.77110

**Published:** 2025-01-07

**Authors:** Mohammed A Jamali, Suad M Abdeen, Thazhumpal C Mathew

**Affiliations:** 1 Department of Psychiatry, Faculty of Medicine, Health Sciences Center, Kuwait University, Kuwait City, KWT; 2 Department of Pathology, Faculty of Medicine, Health Sciences Center, Kuwait University, Kuwait City, KWT; 3 Department of Medical Laboratory Sciences, Faculty of Allied Health Science, Kuwait University, Kuwait City, KWT

**Keywords:** body mass index, fat mass and obesity-associated gene, genetic polymorphism, obesity, rs9969609 polymorphism, single nucleotide polymorphisms, type 2 diabetes

## Abstract

Objective: The aim was to determine the association of the common fat mass and obesity-associated (FTO) gene polymorphism rs9939609 with the risk of developing obesity and type 2 diabetes (T2D) in healthy young university students in Kuwait.

Methods: This cross-sectional study included 201 students from Kuwait University (males = 99; females = 102). The author analyzed the association of FTO and obesity using the body mass index (BMI) as a binary categorical variable (non-obese-BMI < 25 vs. obese-BMI > 25) and the BMI as a continuous variable, using both logistic and linear regression models, respectively. Genotyping of the FTO rs9969609 was performed using the TaqMan single nucleotide polymorphism (SNP) Genotyping Assay (Applied Biosystems, Foster City, USA) and ABI 7500 Fast Real-Time PCR system SDS software (Life Technologies, California, USA) was used for allelic discrimination.

Results: BMI (continuous variable) was not associated with FTO in either the adjusted dominant genetic model (p = 0.33) or the additive model (p = 0.35). Similarly, neither the adjusted dominant (p = 0.66) nor the additive model (p = 0.39) showed FTO as a significant predictor of categorical BMI. In addition, the analysis showed that FTO was not a risk factor for T2D in either the adjusted dominant (p = 0.08) or the additive model (p = 0.17). The FTO allele frequency (%) in the study population (A: 44%; T: 56%) did not significantly differ from the global allele frequency (p-value > 0.05). As for genotype frequency distribution (%) in this study, FTO (AG: 37.8%; AA: 5.5%; GG: 56.7%) was found not to be in equilibrium with the Hardy-Weinberg Equation.

Conclusion: FTO is not a significant predictor of obesity or the future risk of T2D in healthy young adults of Kuwait. The cross-sectional genetic study design is a limitation for the detection or significant predictors of obesity or T2D among young adults in Kuwait.

## Introduction

The International Diabetes Federation (IDF) reported in 2021 that 537 million people, or 10.5% of the global population, had diabetes. The prevalence and incidence of type-2 diabetes (T2D) and its premature mortality and disability rates are growing fast. In 2021, T2D cost $966 billion in healthcare expenses. Diabetes cases are expected to reach 783 million by 2045, with healthcare expenses exceeding $1054 billion. Nearly half of people with diabetes are unaware of their medical condition. T2D has posed economic challenges for healthcare systems worldwide [1]. Farmanfarma et al. determined that the highest prevalence of type-2 diabetes (14.6%) occurred in Middle Eastern countries [2]. Kuwait reports a prevalence of diabetes of more than 21%. The rapidly growing patterns indicate an alarming health situation that requires health policies to overcome the risks [2].

Obesity has become prevalent in the last four decades, e.g., increasing from <1% in 1975 to 6-8% in 2016 among males and females. The percentage has increased from 3% to 11% in men and 6% to 15% in girls [3]. T2D is more frequent in overweight and obese individuals. Ali reported that 70% of overweight and 63% of obese persons had T2D [4]. Weiderpass et al. reported that eight out of 10 Kuwait adults were overweight and obese and recommended immediate public health action in Kuwait [5]. Obesity and T2D are rapidly spreading worldwide and in Kuwait, accounting for 50% prevalence of T2D. Initiatives must be taken by scientists to determine the genetic and environmental causes of obesity [6].

Type-2 diabetes mellitus, the non-insulin-dependent type, is caused by factors including the interaction between genetics, environment, and lifestyle [7]. T2D occurs when the insulin produced from the pancreas cannot maintain blood glucose within normal physiological levels. Insulin in healthy individuals travels via the bloodstream to the liver, muscles, adipose tissues, and other target organs, where it binds on the cell membrane to its tyrosine kinase receptor. After dimerization and autophosphorylation, this ligand-receptor complex will initiate a cascade of intracellular signaling that will eventually lead to increased levels of glucose receptor (GLUT4) on the cell membrane. Insulin causes excess glucose molecules in the blood to be shifted into the cells, restoring blood glucose to its normal physiological levels. T2D occurs when target organs are resistant to insulin and cannot respond properly. Insulin sensitivity reduction is multifactorial and can be attributed to genetics, lifestyle, and environment. Insulin resistance stimulates the beta cells of the pancreas to secrete more insulin to normalize levels of blood glucose. This overactivity will eventually result in the exhaustion and impairment of insulin secretion from the beta cells, resulting in high blood glucose (hyperglycemia) and T2D [8,9].

The fat mass and obesity-associated protein (FTO) gene polymorphism rs9939609 is known to raise the risk of T2D through obesity. The (A;A) genotype makes the risk of developing obesity 3.0 times higher and the risk of T2D 1.6 times higher compared to normal people; however, it only increases the risk of obesity by 2.4 times and the risk for T2D by 1.3 times in individuals with the (A;T) genotype. Conversely, the FTO gene (T;T) genotype lowers the risk of developing T2D and obesity by 1.5 times [10,11]. Among the nuclear proteins found in cells, FTO belongs to the superfamily of nonheme iron oxygenases, including proteins dependent on α-ketoglutarate [12]. To demethylate DNA and RNA, this gene encodes 2-oxoglutarate and Fe (II)-dependent demethylase [8,9]. The expression of the FTO gene is ubiquitous throughout the body and is involved in a variety of organs [13]. The significance of these observations has been further explored with mouse models, which indicate the FTO role in the cardiovascular and nervous systems [11]. Understanding the role of obesity genetics, including FTO, will enable predictions of this association, which in turn may help identify the individuals at risk of obesity due to genetics [10].

Family studies have demonstrated a significant genetic basis to metabolic syndrome, which has been found to cluster in families, and its individual components have been shown to be inherited by offspring as clusters from parents [14,15]. Some of the genetic basis of metabolic syndrome can be explained by the thrifty gene hypothesis, which suggests that these families’ genetic selection switches on energy-conserving genotypes during periods of limited food supply, storing energy as fat rather than glycogen as a genetic defense mechanism against starvation. When families with energy-conserving genotypes are exposed to an abundance of food later in life, they are predisposed to obesity and metabolic syndrome. Low birthweight and intrauterine malnutrition are also risk factors for metabolic syndrome later in life, supporting the thrifty gene hypothesis [16].

Certain single nucleotide polymorphisms (SNPs) were significantly associated with metabolic syndrome and obesity [17]. The FTO gene polymorphism has been linked with the minor allele A of the rs9939609 in the form of the AA genotype. The G:G genotype has also been associated with a risk for obesity and overweight, contributing to metabolic syndrome through obesity and impaired glucose metabolism [18].

The significance of this study is to understand the role of the FTO gene polymorphism to help determine the risk of obesity and T2D among the young people of Kuwait, who are subject to progressively increasing problems of obesity and diabetes. This study aims to determine the association to prevent vulnerable health conditions.

The primary objective is to find the association of the FTO gene polymorphism rs9939609 with the risk of obesity and T2D in healthy young university students in Kuwait.

## Materials and methods

Study design and setting

This cross-sectional study included 201 medical and dental students (males = 99; females = 102) from Kuwait University, Kuwait.

Sample size

A target sample size of 165 was estimated using OpenEpi software, given the 12.2% prevalence of T2D diabetes in Middle Eastern countries as derived from IDF statistics, at a +5% difference and a 95% CI. However, a sample size of 201 was chosen to increase the validity of study.

Inclusion and exclusion criteria

The planned target was to include 200 phenotypically healthy medical and dental students from Kuwait University, 100 males and 100 females. A simple random probability sampling technique was used to anonymously choose those who agreed to sign the consent form, answer the questionnaire, give a blood sample, and complete the anthropometric measurements. The author excluded those who agreed to answer the questionnaire but did not give a blood sample, and vice versa, as well as participants under 21 years old without parental consent.

Data collection

Data collection was conducted with a consent form signed by an adult or parent (see Appendix) provided by each participant in the Pathology Department at the Faculty of Medicine. The consent form clearly illustrated the objectives and benefits of the study, in addition to the risks of the blood collection procedure. It also elaborated on the participant’s rights to exit the study at any time and ensured the confidentiality of the data. The author also clarified in the consent form that the blood samples would be destroyed and not misused following the analysis. Following the consent form, a three-minute synthesized self-designed questionnaire with nine questions (see Appendix) was answered, and anthropometric measurements that included waist circumference, weight, and height were obtained. A blood pressure reading was taken after the participants sat and rested in a private room for 10 minutes as a standardization method.

Licensed phlebotomists from Mubarak Al-Kabeer Hospital (MKH) then drew 12cc of blood from each participant, and the blood samples were immediately labeled with serial numbers that matched the label on the questionnaire for each participant. This was done to protect the participants’ identity and confidentiality. Yellow cap vacutainers were immediately placed in a cold icebox and transferred to the MKH Laboratory for serum analysis of multiple variables on the same day. Lavender cap vacutainers were stored in a -20°C freezer in the Pathology Department Laboratory in the Faculty of Medicine for DNA extraction and SNP genotyping assays in a later phase.

Biochemical analysis of serum samples

Total cholesterol (TC), high-density lipoprotein (HDL)-cholesterol, triacylglycerol (TG), and fasting blood sugar (FBS) were measured using an automated analyzer (DXC 800; Beckman Coulter, Brea, CA, USA). Low-density lipoprotein (LDL)-cholesterol was calculated by the following formula: LDL = (0.97 × TC) - (0.93 × HDL) - (0.19 × TG) [19]. Apolipoprotein B (ApoB) and apolipoprotein A1 (ApoA1) were analyzed by a protein chemistry analyzer (IMMAGE 800; Beckman Coulter, Brea, CA, USA) [20].

DNA extraction and genotyping

The DNA was extracted from 200 µL of whole blood collected in ethylenediaminetetraacetic acid (EDTA)-containing vacutainers using a QIAamp DNA mini kit (Qiagen, California, USA) [21]. Genotyping of the FTO gene polymorphism rs9939609 was performed using a TaqMan SNP Genotyping Assay (Applied Biosystems, Foster City, USA) [22]. ABI 7500 Fast Real-Time PCR system SDS software (Life Technologies, CA, USA) was used to perform and analyze the allelic discrimination [23]. The FTO polymorphism rs9939609 primers used are given in Table 1.

**Table 1:** Primers and probes used in FTO rs9939609 gene sequencing. FTO: fat mass and obesity-associated protein, MGB: minor groove binder.

Primer name	Sequence (5’ to 3’)
Forward primer	5’-GGTTCCTTGCGACTGCT-3’
Reverse primer	5’-AACAGAGACTATCCAAG-3’
MGB probe 1	VIC-GAATTT(A)GTGATGC
MGB probe 2	FAM- GAATTT(T)GTGATGC

Data analysis

Data entry and data analysis were completed using SPSS software v.27 (SPSS, Chicago, IL, USA). The mean, standard deviation (±SD), count (n), and percentage (%) were used to present continuous as well as categorical variables. BMIs were categorized according to the National Institutes of Health classification: underweight ≤18.5, normal weight = 18.5-24.99, overweight = 25-29.99, and obesity ≥30 [24]. A Shapiro-Wilk test was used to assess normality for all variables. P-values were computed using a parametric independent-samples T test and an ANOVA test for HDL, whereas p-values for the remaining variables were computed by a nonparametric Mann-Whitney U test and a Kruskal-Wallis H test, and Pearson’s chi-square test and Fisher’s exact test were used to determine significant differences between genotypic and allelic frequencies in this study as compared to global frequencies obtained from the 1000 Genomes study, with respect to the Hardy-Weinberg equation [25]. Linear regression models were used to predict factors associated with BMI; these factors were represented by an adjusted β-coefficient and a 95% confidence interval (CI), whereas binary logistic regression models were used to predict factors associated with obesity (non-obese vs. obese) and T2D, and these factors were represented by adjusted OR and 95% CI. A p-value less than 0.05 was considered significant.

Ethical statements and funding approval

This article is part of a master’s degree thesis (thesis identification: 0545598) funded and awarded by the College of Graduate Studies of Kuwait University. The thesis is also listed in the National Library of Kuwait (ISBN: 978-99906-1-546-3). The Joint Research Ethical Committee of the Ministry of Health of Kuwait and the Faculty of Medicine of Kuwait University ethically approved the project. The Arabic and English consent forms for adults and minors were also approved by the committee.

## Results

The sample population included 201 healthy young adults, 99 males and 102 females, with an average age of 21 (±2) years old. The highest distribution of the FTO genotypes was TA = 85 (42.3%), followed by TT = 71 (35.3%) and AA = 45 (22.4%). The mean BMI in this study was 25.35 (±0.58) kg/m^2^, and 101 of the study participants (50.2%) had a positive family history (FH) of T2D. Among the anthropometric measurements, waist circumference, SBP, and DBP were significantly higher in the obese group compared to the non-obese group (p-value < 0.05). The mean FBS was higher in the obese group 4.56 (±0.68) in contrast to the non-obese group 4.41 (±0.49), but the difference was not significant (p-value > 0.05). In addition, regarding the lipid profile, Tchol, TG, LDL, and ApoB were significantly higher in the obese group, whereas ApoA1 and HDL were significantly lower (p-value < 0.05; Table 2).

**Table 2:** Anthropometric characteristics, metabolic characteristics, and FTO rs9939609 genotype frequencies, according to obesity (non-obese vs. obese/overweight). BMI: body mass index, BP: blood pressure, FBS: fasting blood sugar, Tchol: total cholesterol, TG: triglycerides, HDL: high-density lipoproteins, LDL: low-density lipoproteins, ApoA1: apolipoprotein A1, ApoB: apolipoprotein B, FH: family history, FTO: fat mass and obesity. *: Indicate Pearson Chi-square value. **: Indicate t-value test statistics.

Criteria	All participants (N = 201)	Non-obese (BMI < 25) (N = 109)	Obese/overweight (BMI ≥ 25) (N = 92)	p-value	Test statistics
Age (years)	21 (±2)	21 (±2)	21 (±2)	0.363	-0.91**
Gender n (%)					
Male	99 (49.3%)	40 (36.7%)	59 (64.1%)	<0.001	15.02*
Female	102 (50.7%)	69 (63.3%)	33 (35.9%)		
FTO genotypes					
TT	71 (35.3%)	38 (53.5%)	33 (46.5%)	0.628	0.55*
TA	85 (42.3%)	49 (57.6%)	36 (42.4%)		
AA	45 (22.4%)	22 (48.9%)	23 (51.1%)		
Mean BMI (kg/m^2^)	25.35 (±0.58)	21.93 (±2.01)	29.41 (±3.87)	<0.001	-16.72**
FH of T2D n (%)					
Yes	101 (50.2%)	56 (51.4%)	45 (48.9%)	0.728	0.12*
No	100 (49.8%)	53 (48.6%)	47 (51.1%)		
Waist circumference (cm)	83 (±14.68)	74.49 (±8.75)	93.08 (±13.9)	<0.001	-11.11**
FBS (mmol/L)	4.48 (±0.58)	4.41 (±0.49)	4.56 (±0.68)	0.158	-1.75**
Systolic BP (mmHg)	118 (±9)	115 (±9)	122 (±9)	<0.001	-5.16**
Diastolic BP (mmHg)	77 (±7)	75 (±7)	78 (±7)	0.002	-3.02**
Tchol (mmol/L)	4.55 (±0.78)	4.37 (±0.75)	4.76 (±0.76)	<0.001	-3.68**
TG (mmol/L)	0.67 (±0.39)	0.59 (±0.34)	0.78 (±0.42)	<0.001	-3.46**
HDL (mmol/L)	1.33 (±0.31)	1.39 (±0.32)	1.26 (±0.29)	0.001	3.07**
LDL (mmol/L)	2.9 (±0.73)	2.69 (±0.67)	3.15 (±0.72)	<0.001	-4.63**
ApoA1 (mg/dL)	1.39 (±0.22)	1.43 (±0.2)	1.35 (±0.24)	0.006	2.54**
ApoB (mg/dL)	0.79 (±0.2)	0.73 (±0.18)	0.87 (±0.2)	<0.001	-5.33**

Analysis of BMIs showed that 101 of the total study participants had normal weight (50.2%), whereas 62 individuals (30.8%) were overweight. Obesity was found in 30 participants (14.9%), and eight were underweight (4%). Obesity and overweight were higher in males, with 18 (18.2%) and 41 (41.4%) respectively, in comparison to females 12 (11.8%) and 21 (20.6%), respectively. A positive FH of T2D was seen in 57 females (55.9%) and 55 males (55.6%; Table 3).

**Table 3:** Categorical BMI and FH of T2D distribution according to gender. FH: family history, T2D: type-2 diabetes.

Criteria, n (%)	All participants (N = 201)	Females (N = 102)	Males (N = 99)	P-value	Pearson Chi-square value
Categorical BMI					
Underweight	8 (4%)	7 (6.9%)	1 (1%)	<0.001	17.35
Normal	101 (50.2%)	62 (60.8%)	39 (39.4%)		
Overweight	62 (30.8%)	21 (20.6%)	41 (41.4%)		
Obese	30 (14.9%)	12 (11.8%)	18 (18.2%)		
FH of T2D					
Yes	101 (50.2%)	57 (55.9%)	44 (44.4%)	0.105	2.63
No	100 (49.8%)	45 (44.1%)	55 (55.6%)		

Mean BMI, categorical BMI, FH of T2D, and FBS did not differ significantly (p-value > 0.05) across FTO genotypes. Anthropometric measurements, lipid parameters, and metabolic characteristics also did not differ with respect to FTO genotypes (p-value > 0.05) (Table 4).

**Table 4:** Relation between FTO rs9939609 genotypes and anthropometric measurements. Shapiro-Wilk test was run for continuous variables to test normality (not shown). All continuous variables showed that data are not normally distributed except Tchol, LDL (p > 0.05); estimated using ANOVA test (indicated p-value by “^”). Data of continuous variables that are not normally distributed underwent the Kruskal-Wallis H test (indicated p-value by “**"). FBS: fasting blood sugar, FH: family history, TG: triglycerides, HDL: high-density lipoproteins, Tchol: total cholesterol, LDL: low-density lipoproteins, ApoA1: apolipoprotein A1, ApoB: apolipoprotein B, FTO: fat mass and obesity. *Categorical variables Chi-square was applied.

Criteria	TT (n = 71)	TA (n = 85)	AA (n = 45)	P-value	Test statistics
Mean BMI (kg/m^2^)	25.22 (±4.69)	25.33 (±5.31)	25.59 (±3.95)	0.776	0.51**
Categorical BMI, n (%)					
Normal ≤24.9	38 (34.9%)	49 (45%)	22 (20.2%)	0.753	1.91*
Overweight 25-29.9	22 (35.5%)	22 (35.5%)	18 (29%)		
Obese ≥30	11 (36.7%)	14 (46.7%)	5 (16.7%)		
FBS (mmol/L)	4.44 (±0.72)	4.48 (±0.5)	4.53 (±0.5)	0.347	2.16**
FH of T2D, n (%)					
Yes	29 (28.7%)	48 (47.5%)	24 (23.8%)	0.303	2.386*
No	42 (42%)	37 (37%)	21 (21%)		
Systolic BP (mmHg)	118 (±9)	118 (±10)	118 (±9)	0.96	2.22**
Diastolic BP (mmHg)	76 (±7)	77 (±7)	77 (±7)	0.81	0.31**
Waist circumference (cm)	83.85 (±13.76)	82.11 (±16.76)	83.34 (±11.79)	0.255	0.64**
Tchol (mmol/L)	4.51 (±0.81)	4.56 (±0.75)	4.57 (±0.81)	0.245	1.42^
TG (mmol/L)	0.73 (±0.42)	0.62 (±0.4)	0.69 (±0.3)	0.036	2.15**
HDL (mmol/L)	1.26 (±0.26)	1.37 (±0.35)	1.35 (±0.3)	0.078	0.60**
LDL (mmol/L)	2.89 (±0.79)	2.91 (±0.68)	2.91 (±0.73)	0.079	2.58^
ApoA1 (mg/dL)	1.37 (±0.19)	1.37 (±0.24)	1.46 (±0.24)	0.058	1.23**
ApoB (mg/dL)	0.81 (±0.22)	0.76 (±0.19)	0.81 (±0.18)	0.203	1.34**

**Figure 1:**
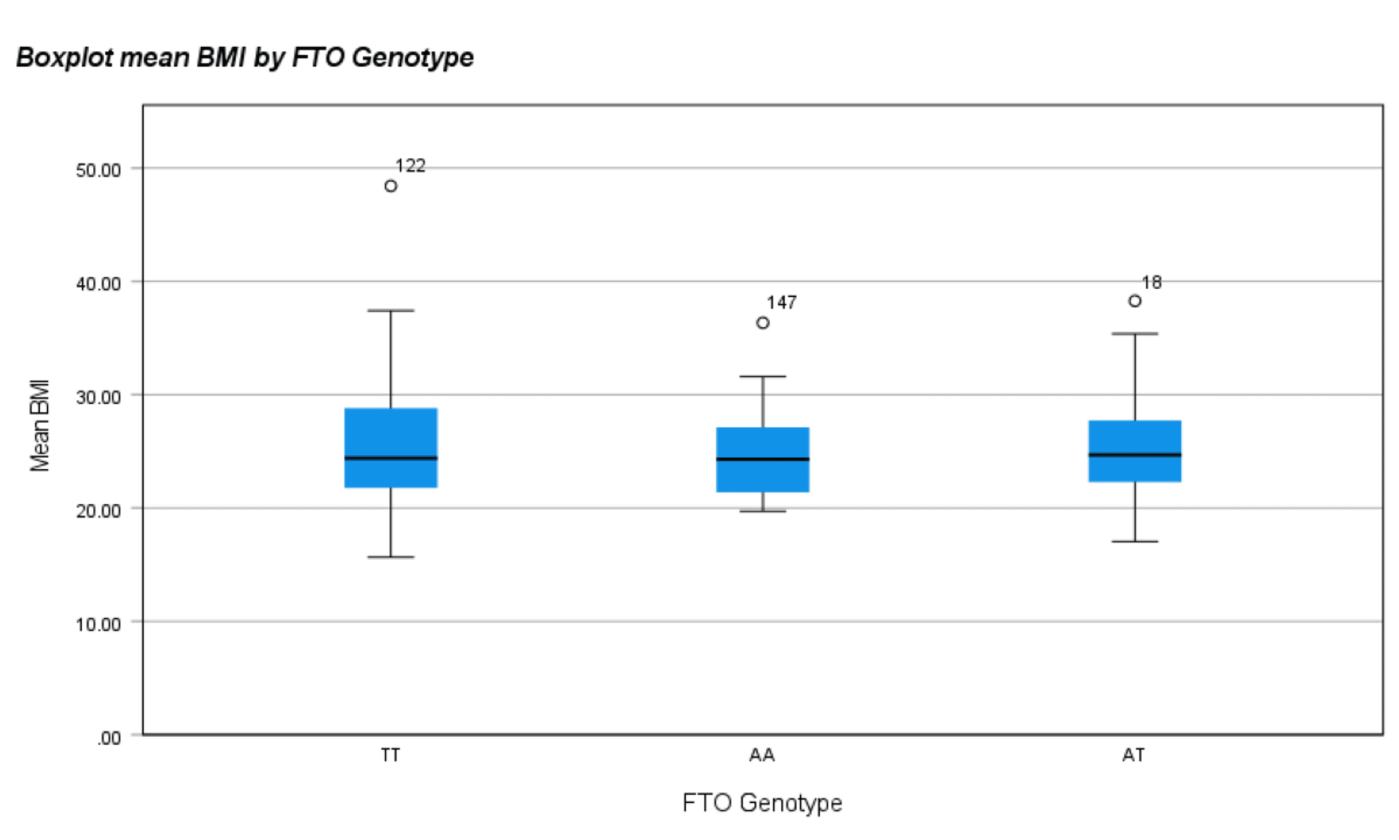
Boxplot showing the distribution of three FTO genotypes (TT, TA, and AA) over mean BMI (weight/height2) with an unadjusted p-value. FTO: fat mass and obesity, BMI: body mass index.

The comparison of the FTO rs9939609 genotype and allele frequencies in this study (observed) to global frequencies (excepted) was obtained from the 1000 Genomes study database [25]. The FTO allele frequency (%) in the study population (A: 44%; T: 56%) did not differ significantly from the global allele frequency (p-value > 0.05). As for the genotype frequency distribution (%) in this study, FTO (AG: 37.8%; AA: 5.5%; GG: 56.7%) was found not to be in equilibrium with the Hardy-Weinberg equation (Table 5). The distribution of three types of FTO gene over mean BMI with an adjusted p = 0.776 is illustrated in Figure 1.

**Table 5:** Comparison of FTO rs9939609 genotype and Allele frequencies in study to global frequencies. *P-values generated using Fisher's exact test. **P-values generated using Pearson's Chi-square test. FTO: fat mass and obesity.

SNP	Genotype frequency			Chi-square (p-value*)	Allele frequency		Fischer exact (P-value**)
	TT	AA	AT		T	A	
FTO	-	-	-	27.46 (0.011)	-	-	8.57 (0.052)
Observed, n (%)	71 (35.3%)	45 (22.4%)	85 (42.3%)		113 (56%)	88 (44%)	
Excepted, (%)	44%	11%	45%		66%	34%	

After adjusting for age and gender, BMI (continuous variable) was not associated with FTO in either the dominant genetic model (p-value = 0.33) or the additive model (p-value = 0.35). Similarly, neither the dominant (p-value = 0.66) nor the additive model (p-value = 0.39) showed FTO as a significant predictor for categorical BMI. In addition, FTO was not a risk factor for T2D in either the adjusted dominant (p-value = 0.08) or the additive model (p-value = 0.17; Table 6).

**Table 6:** Logistic regression models to determine association. FTO additive model = TT vs. TA vs. AA. FTO dominant model = TT vs. TA+AA. Additive and dominant models are adjusted for both gender and age. FTO: fat mass and obesity.

Variable	β	95% CI	p-value
Age	0.03	(-0.34, 0.521)	0.68
Gender	0.31	(1.681, 4.231)	<0.01
FTO (additive model)			
TT	1	-	-
TA	0.69	(-0.842, 2.223)	0.38
AA	0.40	(-0.452, 1.260)	0.35
FTO (dominant model)			
TT	1	-	-
TA+AA	0.07	(-0.677, 2.015)	0.33
Association with BMI (non-obese vs. obese/overweight)	OR	95% CI	p-value
Age	1.09	(0.909-1.301)	0.36
Gender	3.08	(1.732-5.493)	0.01
FTO (additive model)			
TT	1	-	-
TA	1.06	(0.534, 2.087)	0.88
AA	1.18	(0.801, 1.750)	0.39
FTO (dominant model)			
TT	1	-	-
TA+AA	1.15	(0.622, 2.120)	0.66
Association with family history of T2D	OR	95% CI	p-value
Age	0.99	(0.825, 1.178)	0.87
Gender	1.56	(0.890, 2.716)	0.12
FTO (additive model)			
TT	1	-	-
TA	0.56	(0.291, 1.064)	0.08
AA	0.77	(0.528, 1.121)	0.17
FTO (dominant model)			
TT	1	-	-
TA+AA	0.59	(0.325, 1.063)	0.08

## Discussion

FTO and BMI (continuous variables) were not significantly associated with either the dominant genetic model (p = 0.33) or the additive genetic model (p = 0.35) after adjusting for age and gender. Similarly, testing for categorical BMI showed that FTO was not a significant predictor for obesity in either of the adjusted genetic models: the dominant model (p = 0.66) or the additive model (p = 0.39). In respect to T2D, FTO was not reported as a significant risk factor for developing T2D in healthy young adults in the adjusted dominant (p = 0.08) or the additive models (p = 0.17). The prevalence of the high-risk AA genotype was very similar among the non-obese and obese groups.

The association between T2D and FTO was not significant in this study. However, regarding FTO and T2D, the findings contrast with the recently published literature, which reported FTO rs9939609 as a risk factor for hyperglycemia and T2D [26,27]. The findings of this study are contrasted with the existing literature, which increases the range of the topic and the need to explore in-depth whether this difference is due to different population groups (e.g., young vs old or healthy individuals vs individuals with comorbidities) or not. More work is required in the future to validate this finding.

This study reported associations between the AA genotype of FTO and T2D. However, Słomiński et al. found key results in their study that the polymorphism of FTO rs9939609 does not predispose someone to T1D [28]. Moreover, the various genotypes, such as AA, increased the risk of obesity, overweight, and dyslipidemia, and the AT genotype decreased the risk of retinopathy and celiac disease. In sum, FTO gene polymorphism affects overall inflammatory status [28].

A positive family history of T2D and the status of the FTO gene were not found to be associated in this study. The results contrast with the latest pool of findings, such as Amin et al., who meta-analyzed a sample of 26,231 participants with 43,839 controls and found that a positive family history of diabetes plus the minor allele of the FTO gene collectively increased the odds of developing gestational diabetes mellitus two- to three-fold [29]. However, a meta-analysis of subgroups between Asian and non-Asian participants to characterize this association revealed that FTO polymorphism is significantly associated with an increased risk of T2D in Asian subjects with respect to all genetic models, e.g., dominant, recessive, and AC vs CC, except for the US subgroups, in which differences between the AA and AC models were significant. In the Indian population group, the association was not established [29].

Regarding FTO and obesity, results from this study are inconsistent with the latest published literature. Gholamalizadeh et al. found that carriers of the wild-type A allele of the FTO rs9939609 polymorphism have a significantly higher total body fat percentage [30], a finding that is supported by a meta-analysis by Wang et al., which also showed that the minor A allele was significantly linked to higher chances of obesity and metabolic syndrome in Chinese participants [31]. Furthermore, Eghbali et al. carried out a meta-analysis of children and adolescents and showed that the presence of the A allele of FTO rs9939609 is a risk factor for obesity in adolescents and children [32]. Local studies from Kuwait [33] and regional studies from an Arabian population [34], the Emirates [35], Egypt [36], and Turkey [37] were consistent with each other but not with the findings of this study, which indicated no such association. To analyze and widen the geographical coverage of the FTO studies for obesity, the results of this study were compared to global studies, including all the previously mentioned studies, which reported that A-allele carriers of FTO are at a significantly higher risk for obesity, which is opposite to the results of this study. However, Amin et al. showed consistent results in their meta-analysis, which did not find an association between any genetic model of FTO alleles and obesity or T2D [29].

These findings are appraised here because this study reported no association, maybe because of an insufficient sample size (n = 201; males = 99; females = 102); it is possible that a larger sample size is needed to detect any existing genetic association. However, Ağagündüz et al. conducted a study with a similar sample size (n = 200; males = 100; females = 100). The sample size was similar to this study, and both studies followed the Hardy-Weinberg equilibrium [37]. Interestingly, Ağagündüz et al. showed an association between FTO and obesity, in contrast to this study [37]. The reason behind this contrast may lie in the different age groups recruited, given that the mean age (mean ± SD) for Ağagündüz et al. was (39.2 ± 14.01 years) [37] compared to the age group in this study population (21 ± 2 years). Therefore, the current study’s assessment of genetic vulnerability for obesity in age-specific healthy young college medical students could be biased. Moreover, the cross-sectional study design and no follow-up are limitations. This age group is motivated to be involved in healthier lifestyle interventions because they are culturally nominated for marriage. As future medical doctors, they are expected by the local society to be role models for physical fitness. These reasons could motivate the included youthful population to adapt more protective behavioral interventions (which were not assessed in this study) that would partly counteract the genetic susceptibility for obesity, and as a result, this environmental intervention would statistically weaken any genetic association that may occur.

Limitation and generalizability

The study is limited to reporting causal effects due to the cross-sectional study design. To address this limitation, future researchers are encouraged to conduct causal and follow-up studies to reassess obesity and metabolic syndrome components to help detect genetic associations by comparing two different age-specific groups to derive insights. The inconsistent results of this study, especially the comparison of age-specific and ethnicity-specific subpopulations regarding FTO and obesity, are still useful as a reference for future studies and meta-analyses because they represent healthy young adults in Kuwait. This study presents robust evidence that the predisposing gene that may cause type-2 diabetes and obesity showed an insignificant predictor association. The reasons behind this insignificant association may be that the young age, healthy lifestyle, and lack of comorbidities of the medical students indicated the role of the participants’ education.

## Conclusions

The cross-sectional study examined the relationship of the FTO rs9939609 gene polymorphism with obesity, BMI, and T2D in 201 apparently healthy young adults employed in the medical and dental fields. The distribution of the FTO genotype in the study population resembled global allele frequencies but was not in concordance with the Hardy-Weinberg equilibrium. Thus, there was no interaction between sex and FTO genotypes in relation to mean BMI, categorical BMI, or metabolic parameters such as lipid profile and fasting blood sugar. However, males overall had higher mean and categorical BMIs and a higher prevalence of obesity and overweight compared to females. In addition, the significance of the FTO rs9939609 genotype was tested, and it was shown that FTO rs9939609 was not a genetic predictor for BMI, whether continuous and categorical or for T2D, in the context of the additive and dominant effect models. However, gender emerged as a key predictor for obesity. Moreover, anthropometric characteristics, including body mass index and waist circumference, and metabolic factors, including lipid profiles and blood pressure, were higher in obese than non-obese participants, confirming metabolic differences related to BMI. Therefore, efficient gender-sensitive management of metabolic characteristics is considered critical in this sample for obesity-related outcomes. Future researchers should conduct causal and follow-up studies to reassess obesity and metabolic syndrome components to help detect genetic associations by comparing two different age-specific groups to draw insights. The inconsistent results of this study for age-specific and ethnicity-specific subpopulations regarding FTO and obesity are still useful as a reference for future studies and meta-analyses because they represent healthy young adults in Kuwait. This study presents robust evidence that the predisposing gene that may cause type-2 diabetes and obesity did not show a significant predictor association.

## Figures and Tables

**Figure 2:**
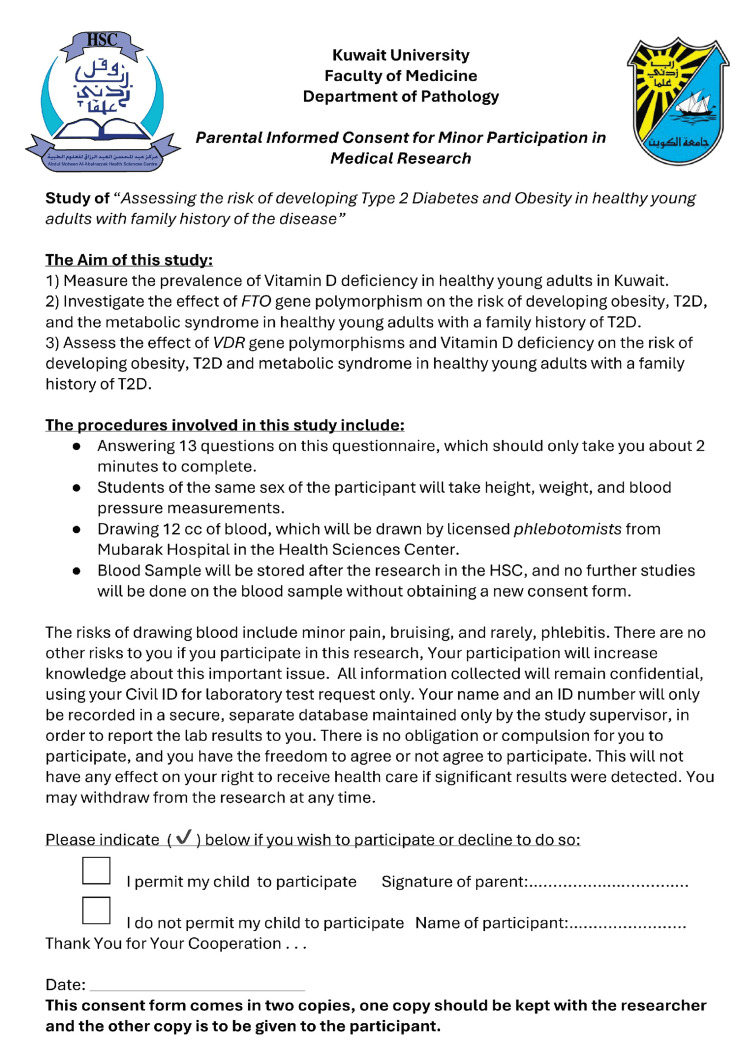
Conset form for adults and minors.

**Figure 3:**
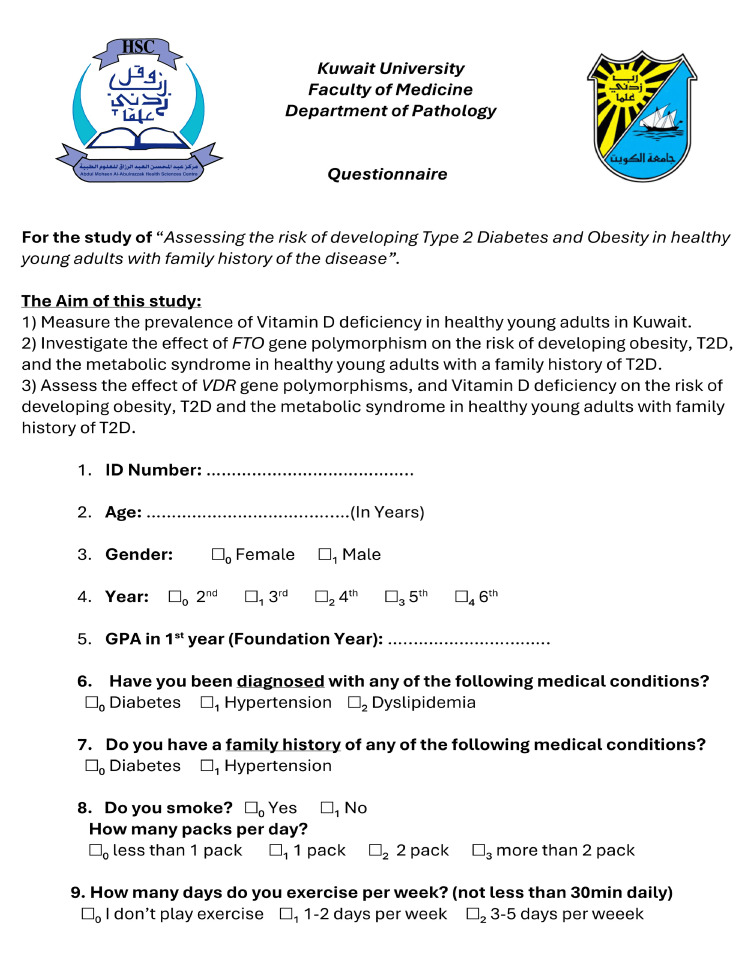
Self-designed questionnaire.
